# Optimization of the CYP inhibition assay using LC-MS/MS

**DOI:** 10.1016/j.mex.2022.101827

**Published:** 2022-08-23

**Authors:** Muhammad Asyraf Abduraman, Nor Hidayah Mustafa, Nik Soriani Yaacob, Azimah Amanah, Mei Lan Tan

**Affiliations:** a School of Pharmaceutical Sciences, Universiti Sains Malaysia, Pulau Pinang 11800, Malaysia; b Drug and Herbal Research Centre, Faculty of Pharmacy, Universiti Kebangsaan Malaysia, Kuala Lumpur 50300, Malaysia; c School of Medical Sciences, Universiti Sains Malaysia, Kubang Kerian, Kelantan 16150, Malaysia; d Malaysian Institute of Pharmaceuticals & Nutraceuticals (IPharm), National Institutes of Biotechnology Malaysia (NIBM), Pulau Pinang 11700, Malaysia

## Abstract

The data presented in this article are related to the research article entitled “Cytochrome P450 inhibition activities of non-standardized botanical products” [1], in which the possible CYP inhibitory properties of botanical products were investigated. This article describes the optimization and bioanalytical method validation of the CYP (Cytochrome P450 inhibition assay) inhibition assays, namely, phenacetin O-deethylase assay, testosterone 6β-hydroxylase assay, felodipine dehydrogenase assay and midazolam 1’-hydroxylase assay using LC-MS/MS.

## Graphical abstract

**Figure:**
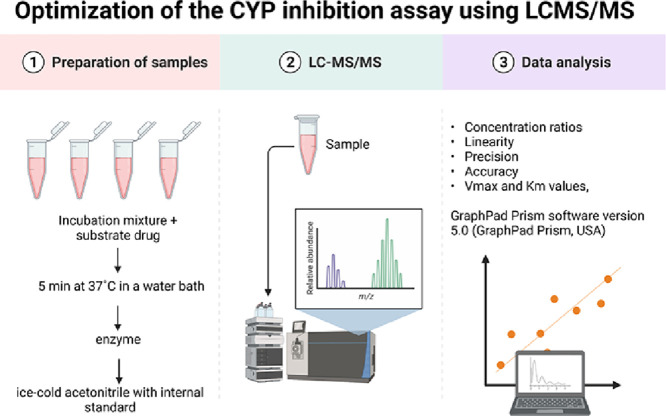

## Specifications table

**Table:** 

Subject area:	Pharmacology, Toxicology and Pharmaceutical Science.
More specific subject area:	Optimization of the phenacetin O-deethylase assay, testosterone 6β-hydroxylase assay, felodipine dehydrogenase assay and midazolam 1’-hydroxylase assay.
Protocol name:	Optimization of CYP Inhibition Assays Using LC-MS/MS analysis.
Reagents/tools:	LC-MS/MS TripleQuadrupole system (Agilent Technologies, Santa Clara, CA, USA) equipped with a 1290 Flexible pump, 1290 FlexCube, 1290 MCT, 1290 Multisampler, 6470 Triple Quadrupole LC-MS (Agilent Technologies, USA) and Agilent MassHunter Workstation Data Acquisition software (Agilent Technologies, USA).
Experimental design:	The retention time, optimum enzyme concentration, incubation time, substrate concentration, Vmax and Km values were determined for phenacetin O-deethylase assay, testosterone 6β-hydroxylase assay, felodipine dehydrogenase assay and midazolam 1’-hydroxylase assay. The bioanalytical method validation was carried out for the LC-MS/MS methods used in CYP inhibition assay.
Trial registration:	N/A
Ethics:	N/A
Value of the Protocol:	• The method is beneficial to researchers who are interested in the drug-drug or herb-drug interaction risks of compounds or herbal compounds. • This data set is beneficial to researchers who want to carry out CYP inhibition assays and bioanalytical method validation in LC-MS/MS methods. • The method is helpful to optimize the CYP inhibition assay and to validate the LC-MS/MS methods.

## Description of protocol

LC-MS/MS analysis and bioanalytical method validation for CYP inhibition assay.

### Determination of retention time

**Fig. 1:**
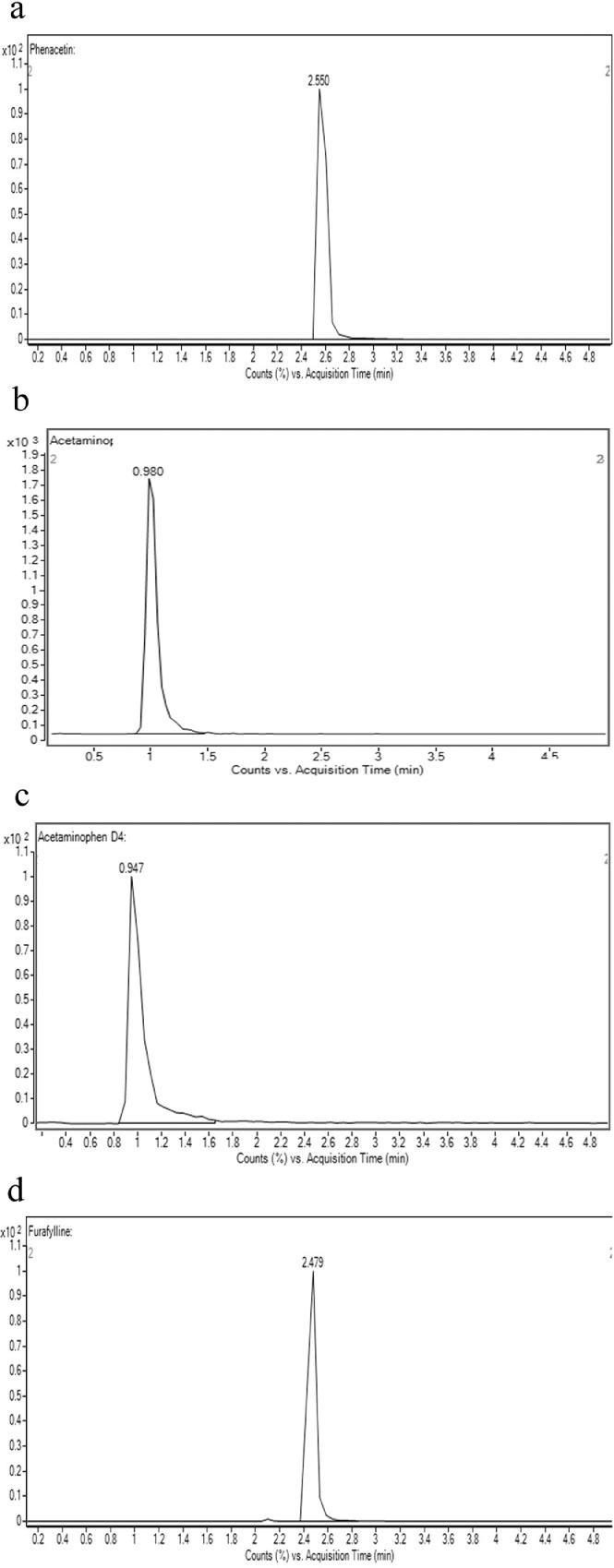

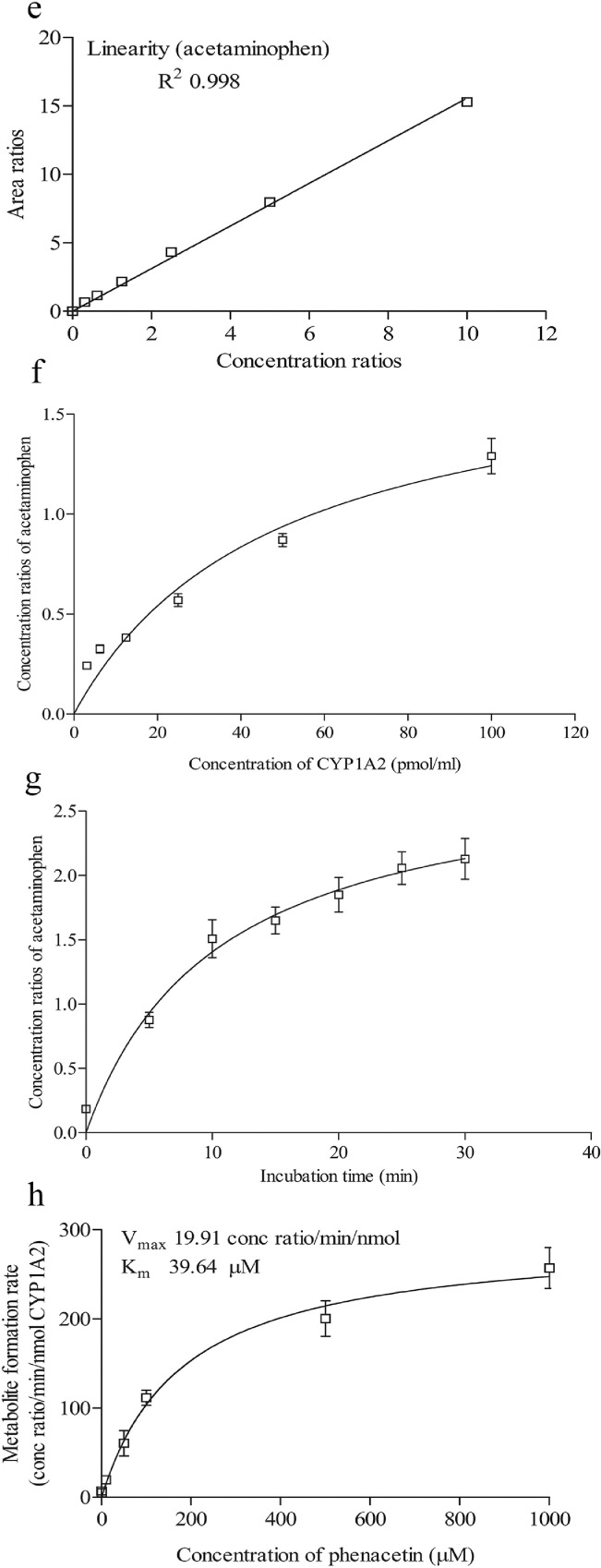
Shows the retention time of (a) phenacetin (substrate; 2.550 min) (b) acetaminophen (metabolite; 0.980 min) (c) acetaminophen D4 (internal standard; 0.947 min) (d) furafylline (CYP1A2 inhibitor; 2.479 min) (e) linearity of acetaminophen standard curve (f) optimization enzyme concentration curve (g) optimization of incubation time curve (h) optimization of substrate concentration (*V*_max_ and *K_m_*) curve. Data are presented as mean ± SD of three independent experiments.

**Fig. 2:**
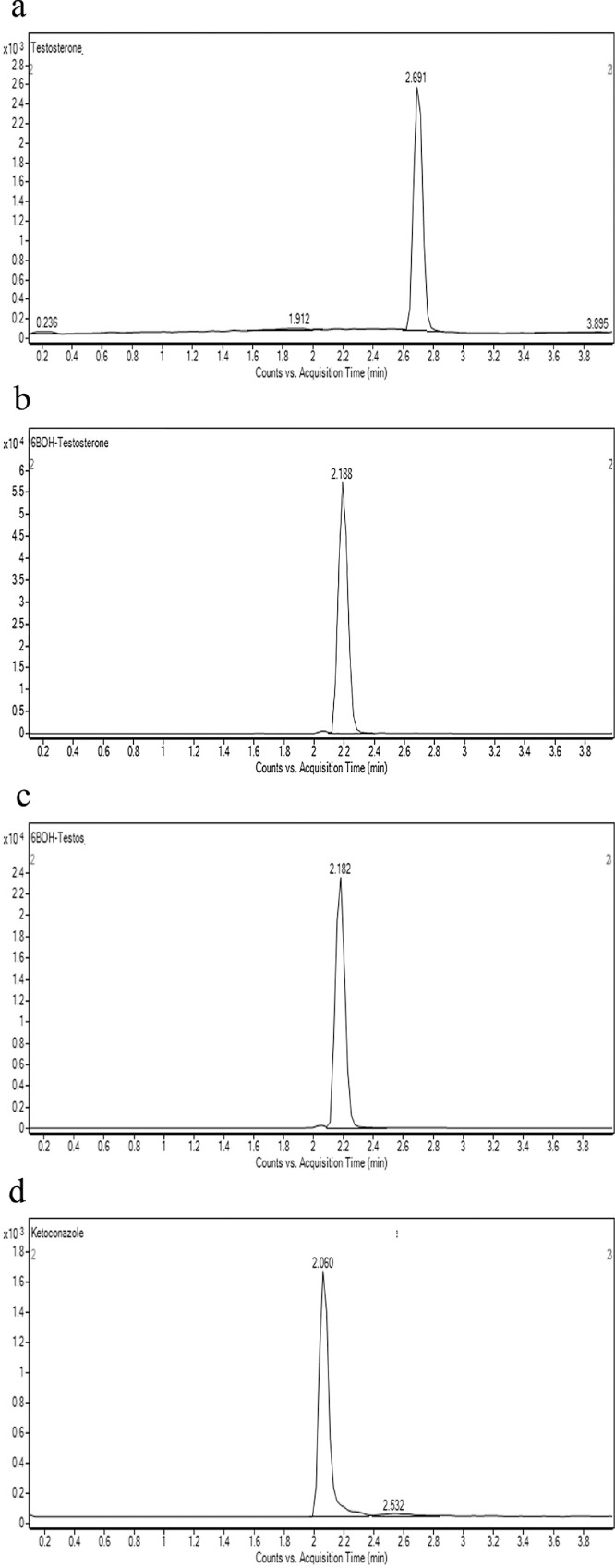

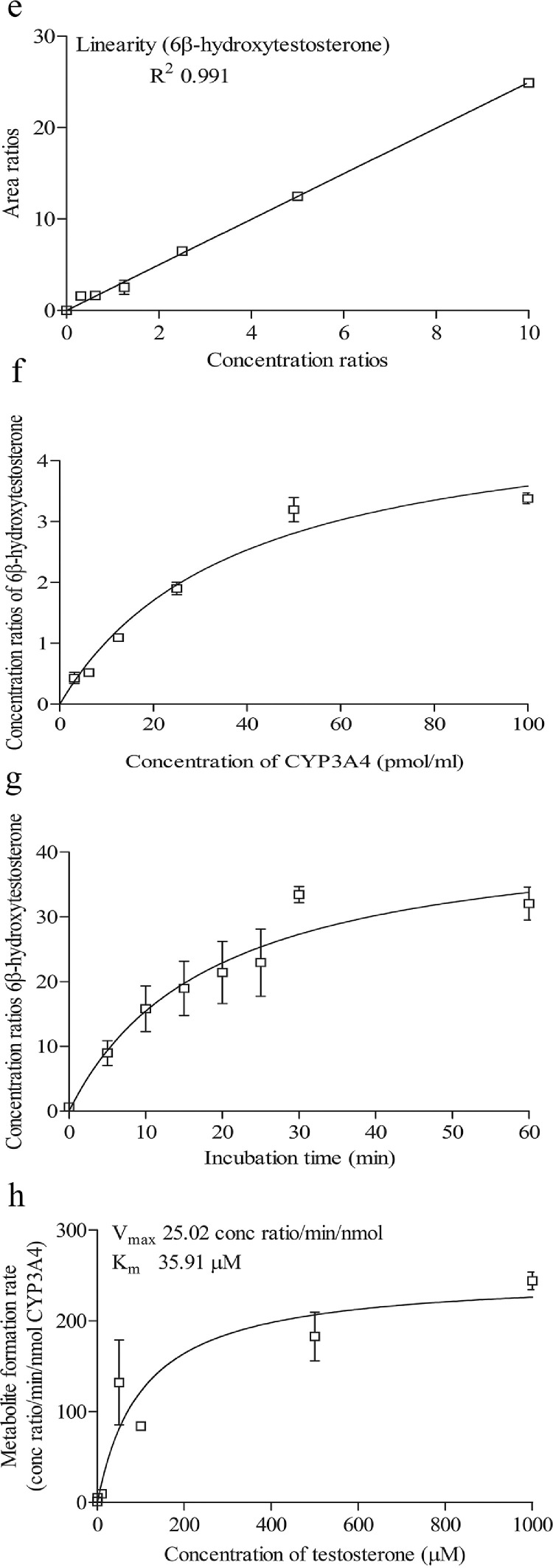
Shows the retention time of (a) testosterone (substrate; 2.691 min) (b) 6β-hydroxytestosterone (metabolite; 2.188 min) (c) 6β-hydroxytestosterone D7 (internal standard; 2.182 min) (d) ketoconazole (CYP3A4 inhibitor; 2.060 min) (e) linearity of 6β-hydroxytestosterone standard curve (f) optimization enzyme concentration curve (g) optimization of incubation time curve (h) optimization of substrate concentration (*V*_max_ and *K_m_*) curve. Data are presented as mean ± SD of three independent experiments.

**Fig. 3:**
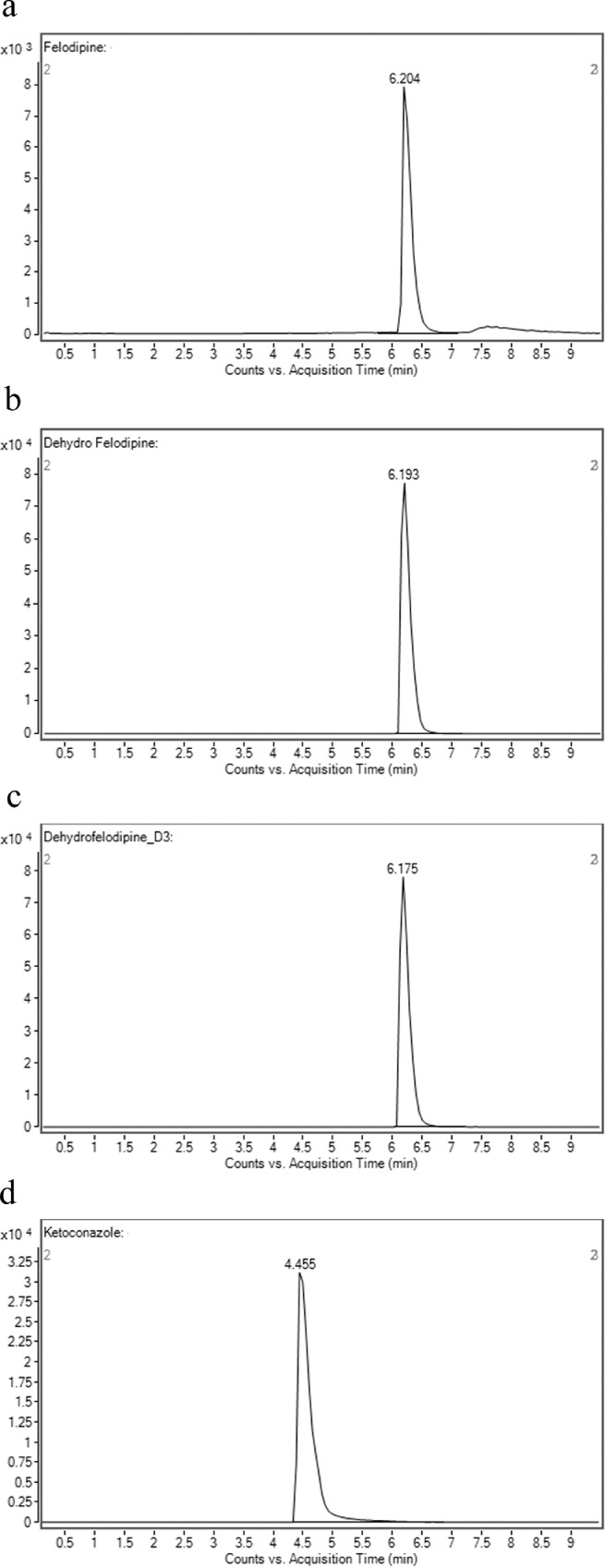

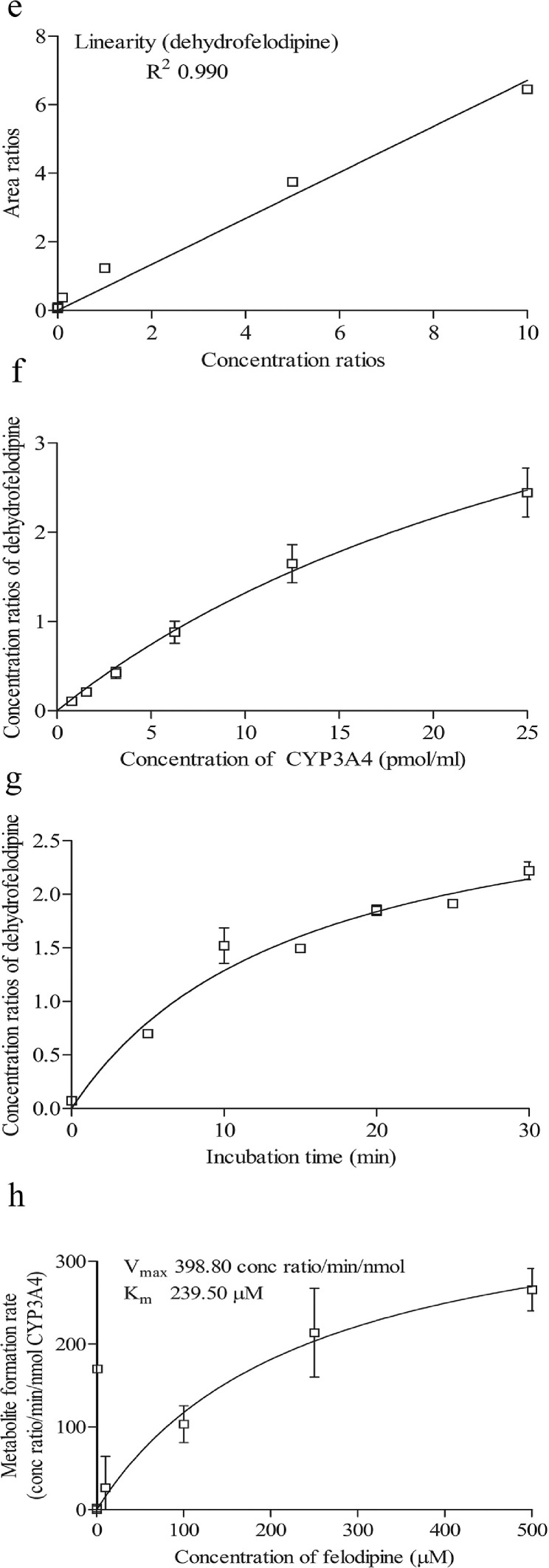
Shows the retention time of (a) felodipine (substrate; 6.204 min) (b) dehydrofelodipine (metabolite; 6.193 min) (c) dehydrofelodipine D3 (internal standard; 6.175 min) (d) ketoconazole (CYP3A4 inhibitor; 4.455 min) (e) linearity of dehydrofelodipine standard curve (f) optimization enzyme concentration curve (g) optimization of incubation time curve (h) optimization of substrate concentration (*V*_max_ and *K_m_*) curve. Data are presented as mean ± SD of three independent experiments.

**Fig. 4:**
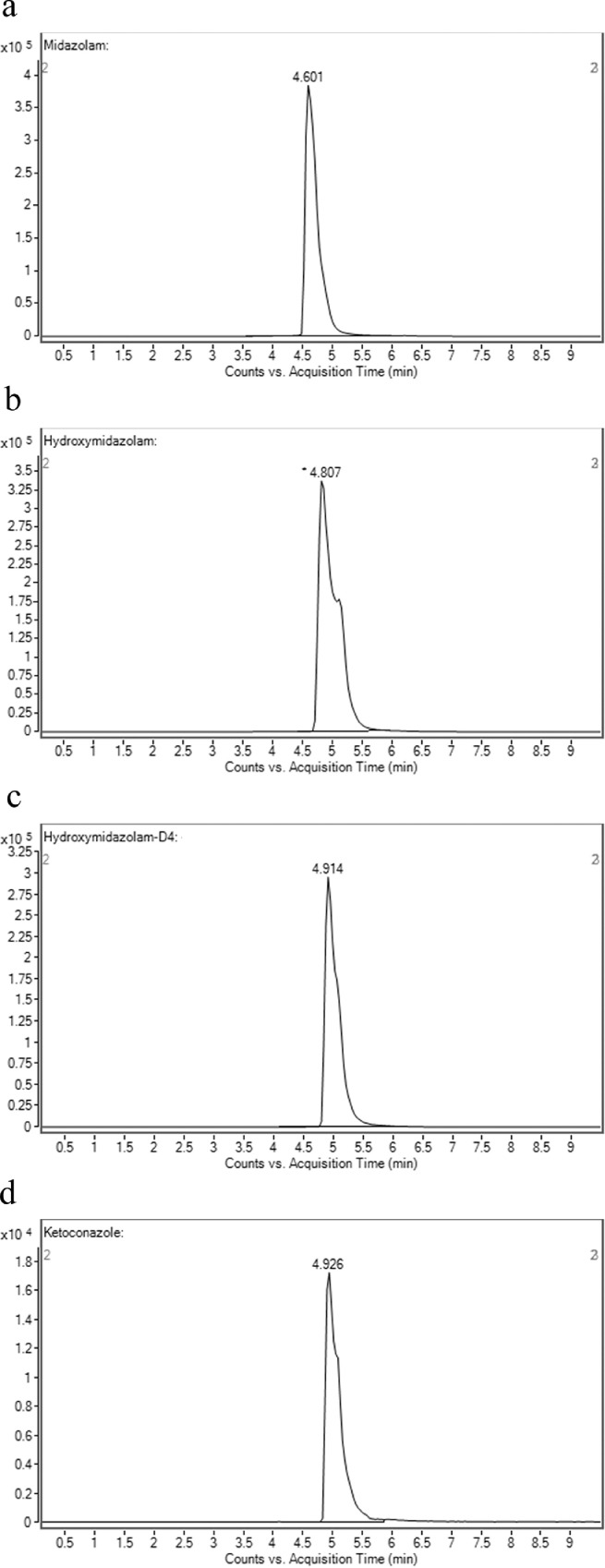

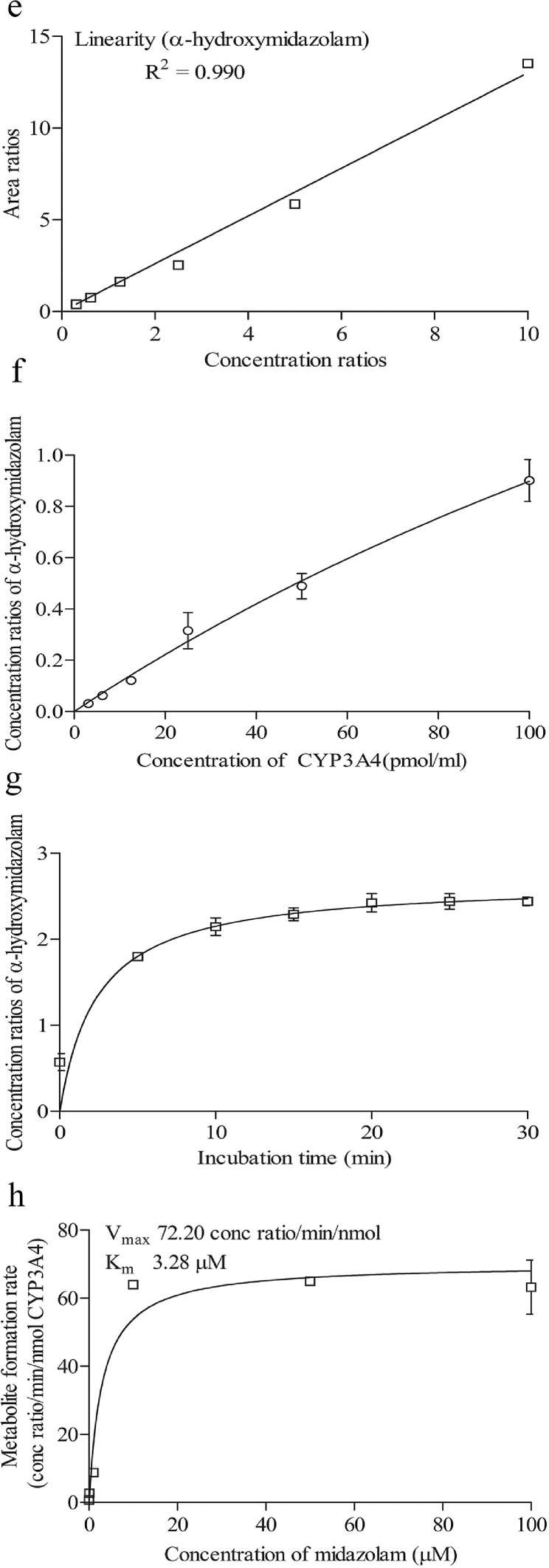
Shows the retention time of (a) midazolam (substrate; 4.601 min) (b) α-hydroxymidazolam (metabolite; 4.807 min) (c) α-hydroxymidazolam D4 (internal standard; 4.914 min) (d) ketoconazole (CYP3A4 inhibitor; 4.926 min) (e) linearity of α-hydroxymidazolam standard curve (f) optimization enzyme concentration curve (g) optimization of incubation time curve (h) optimization of substrate concentration (*V*_max_ and *K_m_*) curve. Data are presented as mean ± SD of three independent experiments.

To determine the retention time of compounds, stock solutions of 1 mM of compounds were first prepared in methanol. 1 µl of stock solution was spiked into an assay incubation mixture containing 0.1 M potassium phosphate buffer, 5.0 µl of NADPH regenerating system solution A [NADP+ and Glc-6-PO₄ (20X)] (Corning, USA), 1.0 µl of NADPH regenerating system solution B [glucose-6-phosphate dehydrogenase (100X concentration)] (Corning, USA) and pure water (Millipore, USA), to yield 10 μM of the compound in a total volume of 100 μl. Samples were first filtered using the 0.22 µM membrane filter and an injection volume of 10 µL was injected into the LC-MS/MS. The retention times of the compounds are shown in Table 1. The chromatograms of compounds are shown in Figs. 1a–d, 2a–d, 3a–d and 4a–d.

**Table 1:** Shows the analytical parameters for the substrate, metabolite and internal standard (IS).

Compound	Retention time (min)	Precursor ion (m/z)	Product ion (m/z)	Fragmentor (V)	Colision energy (eV)
Testosterone 6β-hydroxylase assay (CYP3A4)					
Testosterone	2.691	289.2	97	130	25
6B-Hydroxytestosterone	2.188	305.2	269.1	161	13
6B-hydroxytestosterone-D7	2.182	312.3	276.2	158	13
Ketoconazole	2.084	532.4	490.2	244	33
Felodipine dehydrogenase assay (CYP3A4)					
Felodipine	6.204	385.0	339.1	81	12
Dehydrofelodipine	6.193	383.0	355.1	124	25
Dehydrofelodipine D3 internal standard	6.175	383.0	288.0	124	41
Ketoconazole	4.455	532.4	490.2	195	33
Midazolam 1’-hydroxylase assay (CYP3A4)					
Midazolam	4.601	326.8	292.1	173	45
α-Hydroxymidazolam	4.807	342.8	325.0	136	25
α-Hydroxymidazolam D4	4.914	346.8	329.1	124	41
Ketoconazole	4.926	532.4	490.2	195	33
Phenacetin O-deethylase assay (CYP1A2)					
Phenacetin	2.550	180.1	110.0	121	21
Acetaminophen	0.980	152.1	110.2	124	17
Acetaminophen-D4	0.947	156.2	114.1	102	17
Furafylline	2.479	262.3	81.8	90	25

### Bioanalytical validation

Bioanalytical method validation comprising linearity, between- and within-day precision, and accuracy were determined prior to CYP inhibition assay. To create the linearity curve, a series of concentrations were made for the compounds (metabolite standards) diluted with incubation mixture and finally spiked with internal standard to yield an end concentration of 1 µM. Precision and accuracy were evaluated by analysis of six replicates at various concentrations for within-day calculation and different days for between-day calculation, respectively (Table 2).

**Table 2:** Shows the precision and accuracy data for the metabolites of the CYP inhibition assays using LC-MS/MS analysis.

**Precision and Accuracy**			
Testosterone 6β-hydroxylase assay (6β-hydroxytestosterone)			
End concentration (μM)	Accuracy (% RE)	Precision (% RSD)	
		Intra-day/within day precision or repeatability	Inter-day/between day
10.0	-4.0984	2.0801	3.6740
5.0	18.2315	1.0311	12.5380
2.5	10.7601	1.9087	3.2253
1.25	17.8868	2.5178	13.6569
0.625	5.6896	1.4916	14.5095
0.3125	29.2296	4.5958	28.2393
Felodipine dehydrogenase assay (dehydrofelodipine)			
End concentration (μM)			
Accuracy (% RE)	Precision (% RSD)		
		Intra-day/within day precision or repeatability	Inter-day/between day
10.0	-5.0146	0.6076	4.3075
5.0	19.7797	0.67445	14.3594
1.0	6.8395	1.1322	11.4462
0.1	11.7033	1.3448	10.4196
0.01	-27.8023	3.1468	14.8308
0.001	-1.6381	19.9561	13.6157
Midazolam 1’-hydroxylase assay (α-hydroxymidazolam)			
End concentration (μM)	Accuracy (% RE)	Precision (% RSD)	
		Intra-day/within day precision or repeatability	Inter-day/between day
10.0	3.9194	0.7933	3.8333
5.0	-10.0236	3.14343	11.8240
2.5	-22.0162	1.21813	10.2586
1.25	-0.4095	2.13723	11.0768
0.625	-7.3952	6.76913	15.1256
0.3125	-2.6948	1.96313	17.8183
Phenacetin O-deethylase assay (acetaminophen)			
End concentration (μM)	Accuracy (% RE)	Precision (% RSD)	
		Intra-day/within day precision or repeatability	Inter-day/between day
10.0	6.3512	11.1672	4.6476
5.0	-18.8296	11.5538	13.4528
2.5	-20.2129	16.2585	12.3844
1.25	-19.0570	9.3181	17.6373
0.625	-20.3356	17.3376	17.3059
0.3125	-3.7482	10.9258	28.8720

### Optimization

Briefly, to optimize the incubation time, an incubation mixture containing 5.0 µL of NADPH-regenerating system solution A, 1.0 µL of solution B, pure water, 0.1 M potassium phosphate buffer (pH 7.4) and a specific concentration of substrate drug (10 µM) was prepared in a total volume of 100 µL. The mixture was then incubated at a temperature of 37 ˚C for 5 min. Subsequently, the enzyme activity was started by adding a specific concentration of enzyme (20 pmol/ml) (Corning®SupersomesTM SupersomesEnzyme CYP1A2 Human or Corning®SupersomesTM SupersomesEnzyme CYP3A4 Human), and the mixture was further incubated at 37 ˚C in a water bath for a series of time (5 to 30 min). The enzyme activity was then stopped by adding 100 µL of ice-cold acetonitrile, which contained a final concentration of 1 µM of an internal standard. The mixture was then centrifuged at a speed of 10,000 x g for 5 min at room temperature. Approximately 150 µL of the supernatant was transferred into an auto sampling vial and then subjected to LC-MS/MS analysis. To determine the optimum enzyme concentration to be used in the assay, the preparation of the incubation mixture was similar except that enzyme concentration ranges from 5 to 100 pmol/ml (or 0.781 to 25 pmol/ml for felodipine dehydrogenase assay). Finally, to determine the optimum substrate concentration, a range of substrate concentration was tested up to the maximum of 1 mM.

### LC-MS/MS conditions

Samples were analyzed using Agilent 1290 Infinity II LCMS/MS TripleQuadrupole system (Agilent Technologies, Santa Clara, CA, USA) coupled with a 1290 Flexible pump, 1290 FlexCube, 1290 MCT, 1290 Multisampler and 6470 Triple Quadrupole LC/MS (Agilent Technologies, USA). The substrate and metabolites were separated chromatographically using ZORBAX Extend-C18 Guard column (4.6 mm x 12.5 mm, 5 µm) and ZORBAX Extend-C18 column (3.0 mm x 50 mm, 3.5 µm) (Agilent Technologies, USA), respectively. Eluting compounds were detected in the multiple reaction monitoring (MRM) mode under positive electrospray ionization (ESI+). Other MS conditions included setting the dry gas temperature at 325 °C, the dry gas flow was set to 10 L/min, nebulizer gas pressure was set at 20 psi and capillary voltage was set at 4000 V. For each injection, 10 μL of the sample was injected and peaks were monitored using Triple Quadrupole LC/MS detector (Agilent Technologies, USA) and data acquired using Agilent MassHunter Workstation Data Acquisition software (Agilent Technologies, USA). The samples containing metabolites present in the CYP inhibition assays were identified at specific precursor and product ion production and retention times. The compound-dependent parameters are presented in Table 1.

For phenacetin O-deethylase assay, compounds were identified using a mobile phase consisting of 0.1% (v/v) formic acid in water (A) and 0.1% (v/v) formic acid in methanol (B) and LC-MS/MS analysis based on a linear gradient from an initial 20% (v/v) B to 90% (v/v) B for 3 min followed by column re-equilibration with 20% (v/v) B for 1.5 min at a flow rate of 0.8 ml/min for 7 min [2]. On the other hand, for testosterone 6β-hydroxylase assay, compounds were detected using a similar mobile phase and samples were analyzed using a gradient elution system with a linear gradient from an initial 20% (v/v) B to 90% (v/v) B for 2 min followed by column re-equilibration with 20% (v/v) B for 1.5 min with a flow rate of 0.8 ml/min for 6 min [2]. As for the felodipine dehydrogenase assay, compounds were detected using a gradient elution system with a mobile phase consisting of 0.05% (v/v) formic acid in 5 mM ammonium formate (A) and 0.05% (v/v) formic acid in 95:5 acetonitrile/methanol (B) [3]. A linear gradient analysis with an initial 2% (v/v) B to 40% (v/v) B for 0.5 min followed by column re-equilibration with 98% B for 2.8 min was carried out with a flow rate of 0.5 ml/min for 10 min. Finally, the identification of compounds used in the midazolam 1’-hydroxylase assay was carried out with a mobile phase consisting of 0.1% (v/v) formic acid in water (A) and 0.1% (v/v) formic acid in methanol (B). Briefly, LC-MS/MS analysis was carried out using a linear gradient from 20% (v/v) B to 90% (v/v) B for 5 min followed by 20% (v/v) B for 5 min and at a flow rate of 0.4 ml/min for 10 min. The optimization results are presented in Figs. 1e–h, 2e–h, 3e–h and 4e–h.

### Data analysis

Data were represented as concentration ratios (concentration of compound divided by the concentration of internal standard) and peak ratios (peak AUC of compound divided by peak AUC of internal standard). The unknown concentration ratios of the compound in the sample solution were determined by reference to a standard plot constructed from peak area ratios and concentration ratios of reference compounds. The trendline for the graph was constructed using least-squares linear regression, and the linearity of the standard curve was shown using the coefficient of determination (*R^2^*). The *R^2^* value of 0.9 or better is acceptable linearity within the concentration range. For evaluating the precision of the analytical method, both between- and within-day runs were carried out. Precision was calculated as % relative standard deviation (%RSD), and the precision at each concentration should not exceed 20% of the RSD. The LLOQ was determined by the lowest concentration that can be detected by the LC-MS/MS system with precision and accuracy of less than 20%. Accuracy was determined by calculating the relative error (RE) which is expressed as a percentage, and the mean value at each concentration level should be within 20%. To determine *V*_max_ and *K_m_* values, metabolite formation rate (nmol of metabolite/nmol of enzyme divided by incubation time) were calculated and plotted over substrate concentration and fitted into the least-squares Michaelis Menten model using GraphPad Prism software version 5.0 (GraphPad Prism, USA) [4]. The parameters used to generate the Michaelis Menten curve was based on GraphPad Prism software whereby the *V_max_* and *K_m_* value were set at no constraint type and greater than 1. The model equation was based on Y=V_max_*X/(*K_m_*+X) whereby the software automatically generates the Lineweaver-Burke plot corresponding to the nonlinear regression fit and subsequently generate the Vmax and Km based on best fit values. Goodness of fit was based on parameters such as R squared (R2) > 0.90 and degrees of freedom.

## Ethics statements

There is no ethical statement relating to this data.

## CRediT authorship contribution statement

**Muhammad Asyraf Abduraman:** Methodology, Formal analysis, Data curation. **Nor Hidayah Mustafa:** Methodology, Formal analysis, Data curation. **Nik Soriani Yaacob:** Resources, Conceptualization, Validation, Writing – review & editing. **Azimah Amanah:** Methodology, Formal analysis, Data curation. **Mei Lan Tan:** Methodology, Formal analysis, Data curation, Resources, Conceptualization, Validation, Writing – review & editing.

## Declaration of Competing Interest

The authors declare that they have no known competing financial interests or personal relationships that could have appeared to influence the work reported in this paper.

## Data Availability

Data will be made available on request. Optimization of the CYP inhibition assay using LC-MS/MS analysis (Original data) (Mendeley Data).
